# Oxia Planum: The Landing Site for the ExoMars “Rosalind Franklin” Rover Mission: Geological Context and Prelanding Interpretation

**DOI:** 10.1089/ast.2019.2191

**Published:** 2021-03-10

**Authors:** Cathy Quantin-Nataf, John Carter, Lucia Mandon, Patrick Thollot, Matthew Balme, Matthieu Volat, Lu Pan, Damien Loizeau, Cédric Millot, Sylvain Breton, Erwin Dehouck, Peter Fawdon, Sanjeev Gupta, Joel Davis, Peter M. Grindrod, Andrea Pacifici, Benjamin Bultel, Pascal Allemand, Anouck Ody, Loic Lozach, Jordan Broyer

**Affiliations:** ^1^ Univ Lyon, Univ Lyon 1, ENS Lyon, CNRS, LGL-TPE, 2 Rue Raphael Dubois, F-69622 Villeurbanne, France, France.; ^2^ Institut d'Astrophysique Spatiale, Univ Paris Sud, CNRS, UMR 8617, Univ Paris-Saclay, Bat 120-121, F-91405 Orsay, France.; ^3^ Open Univ, Dept Earth & Environm Sci, Milton Keynes MK7 6AA, Bucks, England.; ^4^ Univ London Imperial Coll Sci Technol & Med, Dept Earth Sci & Engn, London SW7 2AZ, England.; ^5^ Department of Earth Sciences, Natural History Museum, London, United Kingdom.; ^6^ Univ G DAnnunzio, IRSPS, I-65127 Pescara, Italy.; ^7^ Department for Geosciences, Centre for Earth Evolution and Dynamics (CEED), University of Oslo, Oslo, Norway.

**Keywords:** Landing site, Mars, ExoMars, Oxia Planum.

## Abstract

The European Space Agency (ESA) and Roscosmos ExoMars mission will launch the “Rosalind Franklin” rover in 2022 for a landing on Mars in 2023.The goals of the mission are to search for signs of past and present life on Mars, investigate the water/geochemical environment as a function of depth in the shallow subsurface, and characterize the surface environment. To meet these scientific objectives while minimizing the risk for landing, a 5-year-long landing site selection process was conducted by ESA, during which eight candidate sites were down selected to one: Oxia Planum. Oxia Planum is a 200 km-wide low-relief terrain characterized by hydrous clay-bearing bedrock units located at the southwest margin of Arabia Terra. This region exhibits Noachian-aged terrains. We show in this study that the selected landing site has recorded at least two distinct aqueous environments, both of which occurred during the Noachian: (1) a first phase that led to the deposition and alteration of ∼100 m of layered clay-rich deposits and (2) a second phase of a fluviodeltaic system that postdates the widespread clay-rich layered unit. Rounded isolated buttes that overlie the clay-bearing unit may also be related to aqueous processes. Our study also details the formation of an unaltered mafic-rich dark resistant unit likely of Amazonian age that caps the other units and possibly originated from volcanism. Oxia Planum shows evidence for intense erosion from morphology (inverted features) and crater statistics. Due to these erosional processes, two types of Noachian sedimentary rocks are currently exposed. We also expect rocks at the surface to have been exposed to cosmic bombardment only recently, minimizing organic matter damage.

## 1. Introduction

The scientific objectives of the European Space Agency (ESA)–Roscosmos ExoMars mission are primarily to search for signs of past and present life on Mars, then to investigate the aqueous/geochemical environment as a function of depth in the shallow subsurface and to characterize the surface environment (Vago *et al.*, 2015a). The ExoMars rover, recently named after Rosalind Franklin (1920–1958), will carry a suite of instruments—the Pasteur payload—dedicated to geology and astrobiology. It will be able to travel several kilometers during its nominal lifetime of 218 sols (Vago *et al.*, 2015a). The Pasteur payload (Barnes *et al.*, 2006) features a suite of instruments operating at all scales: (1) a mast-mounted panoramic instrument: wide-angle and high-resolution multispectral visible wavelength cameras, and near infrared spectrometer; (2) a chassis-mounted ground-penetrating radar and neutron detector; (3) a subsurface drill capable of reaching a depth down to 2 m to collect specimens with associated contact instruments for studying rocks and collected samples, a closeup imager and an infrared spectrometer in the drill head; (4) a sample preparation and distribution system; and the analytical laboratory, the latter including a visible and near infrared microimaging spectrometer, a Raman spectrometer, and a gas chromatograph mass spectrometer (*e.g.*, Vago *et al.*, 2017). The ability to drill down to 2 m depth is needed to maximize the chance of reaching well-preserved organic molecules that are less affected by cosmic radiation and surface oxidants (Vago *et al.*, 2017).

From a science requirement perspective with respect to the scientific payload of ExoMars, the selected landing site was required to be ancient, from the early Mars period (Noachian to early Hesperian, >3.6 Ga), during which, as many studies indicate, abundant water-related activity likely occurred (Carr and Head, 2010). The site must bear abundant morphological and mineralogical evidence of a long-lived aqueous activity and expose sedimentary rocks that are good candidates for organic matter preservation. More importantly, the relevant outcrops must be widely distributed across the landing ellipse to ensure that at least some suitable rock targets will be accessible within the traverse range (of the order of a few km maximum), wherever the rover lands within its large landing ellipse of ∼19 by 120 km (Vago *et al.*, 2017).

**FIG. 1.**
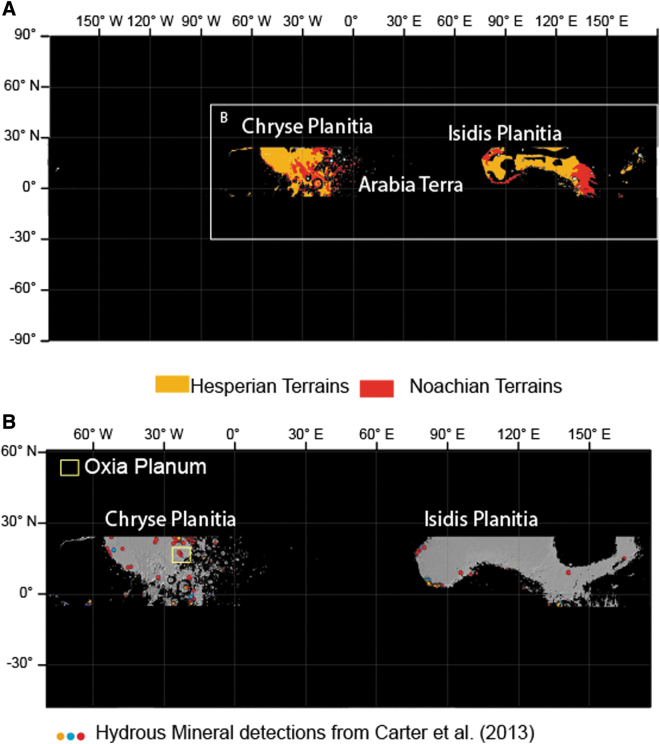
**(A)** Global map darkening the places noncompliant with elevation, thermal inertia, and latitude constraints. Hesperian is in orange units and Noachian is in red (Tanaka *et al.*, 2014). **(B)** Enlarged map focused on Chryse and Isidis regions of Mars darkening the places noncompliant with elevation, thermal inertia, and latitude constraints and highlighting the hydrous mineral detection from Carter *et al.* (2013).

To meet the scientific goals and minimize the risk for landing, a 5-year landing site selection process (2014–2018) has been conducted by ESA, during which eight candidate sites were down selected to one: Oxia Planum. In this article, we present our approach to finding a place on Mars that would meet both the engineering constraints and the scientific objectives, and how this led us to the Oxia Planum site, a wide clay-bearing plain located between 16° and 19° N and −23° to −28° E (Fig. 1).

Here, we present preliminary geological observations of the Oxia Planum site that show the region to have had a diverse aqueous history. This article is accompanied by a second article that focuses on the mineralogy and the geochemistry of the Oxia Planum region, and discusses aqueous alteration scenarios and their relevance to mission objectives. This article details the different geological units of the region in chronological order and ends with a discussion of prelanding interpretation of the geological evolution of the region from the perspective of the ExoMars mission.

## 2. Data Sets and Methods

A large data set has been processed. All the processed data from regional to local scales are available to the community through the MarsSI facility (Quantin-Nataf *et al.*, 2018) [https://marssi.univ-lyon1.fr/MarsSI/].

### 2.1. Global data sets

We use Geographic Information System (GIS) software (ESRI/ArcGIS) to combine a large set of data, including Mars Orbiter Laser Altimeter (MOLA) for topography (Smith *et al.*, 2001), Thermal Emission Imaging System (THEMIS) infrared daytime and night-time global mosaics (Christensen *et al.*, 2004; Edwards *et al.*, 2011), global maps of thermal inertia derived from Thermal Emission Spectrometer (TES) data (Putzig *et al.*, 2005), the global geological map of Mars (Tanaka *et al.*, 2014), Observatoire pour la Mineralogie, l'Eau, les Glaces et l'Activité (OMEGA) mafic mineral global maps (Ody *et al.*, 2012), and hydrated minerals global maps derived from OMEGA/Compact Reconnaissance Imaging Spectrometer for Mars (CRISM) data (Carter *et al.*, 2013). For regional analyses of the Oxia Planum area, we also use THEMIS-based thermal inertia, which has a better spatial resolution than TES-based thermal inertia (Fergason *et al.*, 2006; Christensen *et al.*, 2013).

### 2.2. Image data

We used regional-scale imaging data, including High Resolution Stereo Camera (HRSC) (Jaumann *et al.*, 2007) and Context Camera (CTX, 5–6 m/pixel) (Malin *et al.*, 2007). We added local-scale data such as High Resolution Imaging Science Experiment (HiRISE, 25–50 cm/pixel) (McEwen *et al.*, 2007) images, including both color and RED-filter data products (Delamere *et al.*, 2009). All HiRISE and CTX images were downloaded from the NASA Planetary Data System (PDS) server and then map-projected by the MarsSI facility (Quantin-Nataf *et al.*, 2018). The full list of images used in this article is provided in Supplementary Table S1, and the HiRISE coverage of Oxia Planum is presented in Supplementary Fig. S1.

### 2.3. Topographic data

We collected or computed all digital elevation models available from HRSC, CTX, and HiRISE stereopairs. HRSC Digital Terrain Models (DTMs) were downloaded from the ESA Planetary Science Archive server (Gwinner *et al.*, 2010). CTX DTMs were produced by using the NASA Ames stereo pipeline (Beyer *et al.*, 2018) in the MarsSI platform (Quantin-Nataf *et al.*, 2018). HiRISE DTMs were produced with the SOCET SET software following the method presented in the work of Kirk *et al.* (2009). We were able to produce complete coverage of the landing site study area with CTX DEMs and images and, by the end of the landing site selection process, almost complete coverage of the study area with HiRISE images. HiRISE DEMs covered a much smaller part of the study area. The DTMs used in the article are listed in Supplementary Table S2.

## 3. ExoMars Landing Constraints and the Identification of Oxia Planum as a Potential Landing Site

To search for an appropriate landing site for the ExoMars rover, we first considered the numerous criteria for a safe landing and successful operations. The first criterion is the latitude, to avoid areas of Mars with extreme temperatures and maximize solar power; the ExoMars platform and rover are designed to operate only at latitudes that range from 5°S to 25°N. Then, the elevation must be lower than −2 km relative to MOLA reference to provide a thick enough atmospheric column to slow down the landing module for safe entry, descent, and landing. The area must also expose *in situ* bedrock (as opposed to unconsolidated sediments or float rocks); here, we used a threshold thermal inertia value of 150 thermal inertia units (TIU) (TIU are J · m^−2^·s^−0.5^·K^−1^, Putzig *et al.*, 2005) to identify rocky surfaces. Then, the ellipse has to be placed to avoid high slopes, which translates at the MOLA subkilometer scale into avoiding slopes >8°.

The first step of our search for a suitable landing site consisted of combining the engineering and scientific constraints that can be addressed at a global scale, such as the latitude constraints, the elevation range, the thermal inertia, and the age of martian geological units. This step was achieved by using GIS, and the results are presented in Fig. 1. Figure 1 shows areas in black that are outside the 5°S to 25°N latitude band, higher than −2 km with respect to the MOLA Geoid regions with thermal inertia <150 TIU, and any Amazonian terrains as mapped by Tanaka *et al.* (2014). The process identified two “windows” that fit the criteria of latitude, elevation, thermal inertia, and age as follows: (1) Chryse Planitia and the western end of Arabia Terra, and (2) Isidis Planitia and the circumferential area of Elysium Mons. Within these two windows, dissected terrains of Noachian age are present mainly on the border of the Chryse and Isidis basins, near the martian dichotomy boundary.

The second step of our approach was to search for signs of past aqueous activity in the mineralogical record. To this end, we overlaid the global OMEGA and CRISM hydrous minerals map produced by Carter *et al.* (2013) onto the map showing the two windows described above (Fig. 1B). The combined map shows that hydrous minerals are present in several places on the border of both Chryse and Isidis basins. We then checked the various detections of hydrous minerals and, where possible, associated them with surface morphology to calculate the spatial extent of these deposits. We compared these with the size of the ExoMars landing ellipse (∼19 by 120 km). These criteria highlighted Oxia Planum in particular, as a wide plain rich in hydrated minerals (mostly Fe/Mg clays, see below), which appears relatively smooth compared with other regions on Mars. We proposed to name this site “Oxia Planum” because it is a plain region located inside the Oxia quadrangle. Oxia Planum was approved in 2019 as an official feature of the martian surface by the International Astronomical Union (IAU) Working Group for Planetary System Nomenclature.

**FIG. 2.**
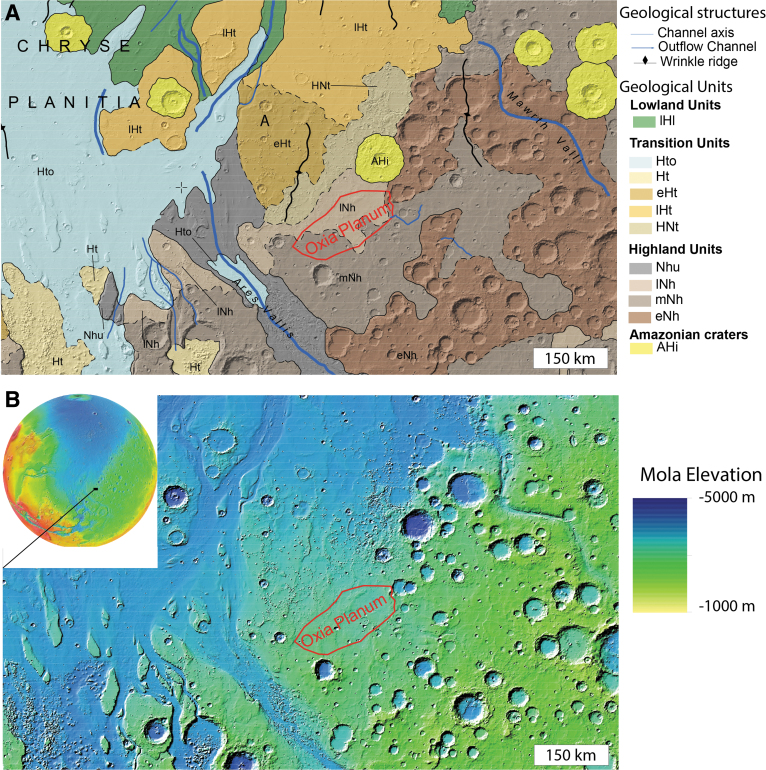
Location of Oxia Planum area in the region between Ares Vallis and Mawrth Vallis. **(A)** Regional geological map from the work of Tanaka *et al.* (2014). **(B)** Topographic context of MOLA data (Smith *et al.*, 2001). MOLA, Mars Orbiter Laser Altimeter.

**FIG. 3.**
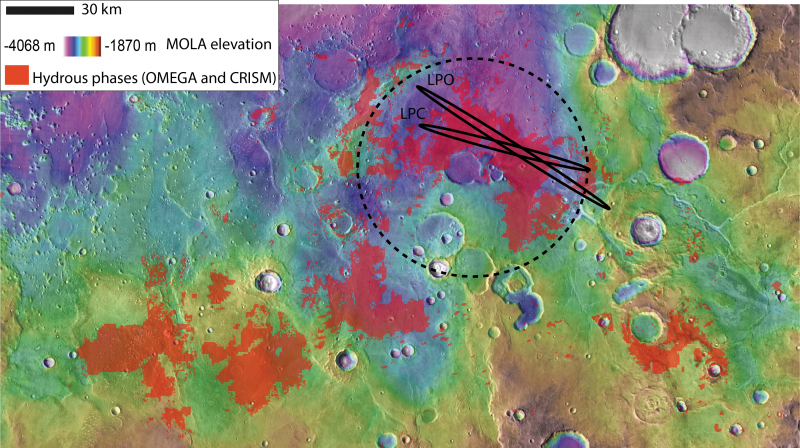
Hydrous mineral map from OMEGA and CRISM MSP data (Carter *et al.*, 2016) over MOLA elevation map. The hydrous minerals bearing unit is widespread and drapes the current topography from −2600 m of elevation to −3100 m where ExoMars 2020 ellipses are located. The dark ellipses are the 3-sigma probability of the two ellipse endmembers of the launch period: LPO and LPC. The dotted circle highlights a putative ancient circular depression. CRISM, Compact Reconnaissance Imaging Spectrometer for Mars; OMEGA, Observatoire pour la Mineralogie, l'Eau, les Glaces et l'Activité; LPC, launch period closing; LPO, launch period opening; MSP, multispectral survey.

Oxia Planum is located between 16° and 19° N and −23° to −28° E (Fig. 2), on the eastern border of Chryse Planitia. The region is located between two outflow channel systems: Mawrth Vallis to the NE and Ares Vallis to the SW. The region with clay mineral detections straddles two geological units as mapped by Tanaka *et al.* (2014), both of which are highlands bedrock units: the Middle Noachian highland unit (mNh) and the Late Noachian highland unit (lNh). Both units are crosscut by major wrinkle ridges and major channels such as Coogoon Valles, a large valley system that has its outlet in Oxia Planum. As shown by the geological and topographic maps of Fig. 2, the clay-bearing region is characterized by relatively smooth topography and contains fewer impact craters than nearby early Noachian terrains such as the region south of Mawrth Vallis. The ExoMars landing ellipse we proposed for Oxia Planum is located in the northern part of the widespread Oxia clay-bearing region, at the outlet of Coogoon Valles system (Molina *et al.*, 2017) (Fig. 3). Accordingly, the rest of the article will focus on the geology of the final landing site ellipse area. In Fig. 3, both 3-sigma launch period opening (LPO) and launch period closing (LPC) ellipses considered for the launch in 2020 are displayed. At the time of writing, the landing ellipses for the 2022 launch have not been finalized, but indications are that the ellipse size and azimuth will be similar to those planned for 2020 (J. Vago, 2020, pers. comm.). This specific location was chosen because of the presence of a large-scale basin that is described below.

## 4. The Geology of the Oxia Planum ExoMars Rover Landing Site

**FIG. 4.**
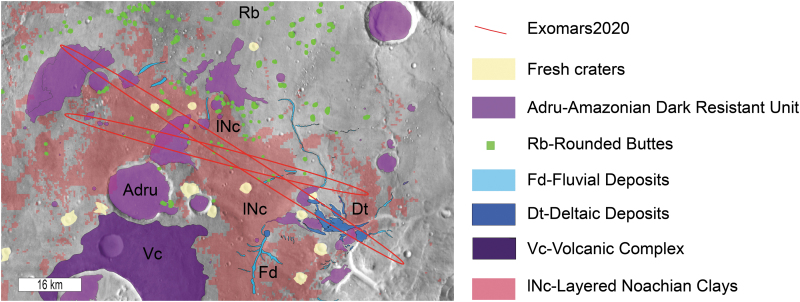
Geological map of the ExoMars ellipses area. The red ellipses are the three-sigma LPO and LPC ellipses.

Figure 4 summarizes the mapping of the geological units identified and described in the following subsections.

### 4.1. The Noachian layered clay-bearing unit (lNc)

**FIG. 5.**
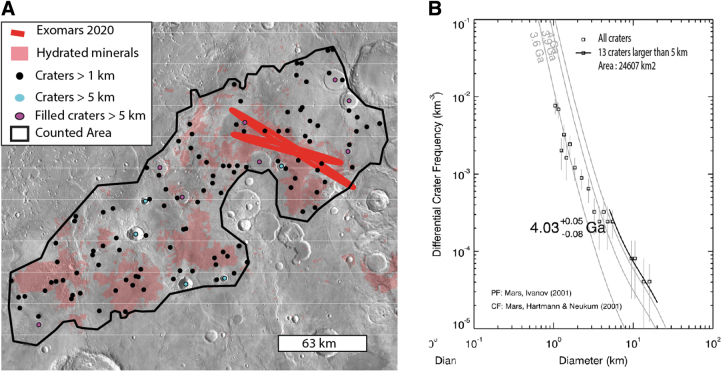
**(A)** Location of the impact craters used in **(B)** for the crater-based age determination, **(B)** cumulative crater density superimposed on the clay-bearing unit in an incremental diagram performed by the Craterstats2 software (Michael *et al.*, 2012). In the crater size distribution of craters >1 km, only the craters >5 km follow an isochron. Only these craters >5 km have been used to assess the age of the clay-bearing unit. The size distribution of the crater <5 km has a lower slope than any isochron (*e.g.*, some isochrons are plotted in light gray), indicating continuous resurfacing processes (see section “Erosional history of Oxia Planum”). We can note that some of the craters are partly filled (marked in pink) by the dark resistant unit described latter in section “Dark resistant unit (Adru).” For these filled craters, we carefully checked that the clay-bearing unit is exposed in the ramparts of the filled craters attesting that the craters were emplaced in the clay-bearing rocks.

The hydrated minerals of Oxia Planum were mapped by using OMEGA and CRISM data based on absorption bands centered at 1.9 and 2.3 microns according to a methodology detailed in the work of Carter *et al.* (2016). The combination of absorption bands centered at these wavelengths is typical of Fe/Mg clays. In the case of Oxia Planum, the dominant spectra match that of a vermiculite-smectite type of clay (Carter *et al.*, 2016). As shown in Fig. 3, the extended clay-bearing unit ranges in elevation from −2600 m in the southwest part of Oxia Planum to −3100 m where the ellipses are located and thus appears to have overlain the pre-existing topography, in a similar way to the clays observed in Mawrth Vallis region (Loizeau *et al.*, 2012). According to the global geological map (Tanaka *et al.*, 2014), the unit overlaps terrains of mid- and late Noachian age (Fig. 2), so the unit is mid-Noachian or younger. Assessment from size distribution of craters from 1 to 120 km in diameter over the entire clay-bearing unit of Oxia Planum returns an age of ∼4 Ga. Ages have been derived by fitting a crater production function from Ivanov (2001) and by using the chronology function from the work of Hartmann and Neukum (2001) to translate relative crater frequencies into absolute model ages, in agreement with a mid-Noachian age (Fig. 5). This mid-Noachian age is based on the craters >5 km whose distribution aligns along an isochron in a size frequency diagram (Fig. 5).

**FIG. 6.**
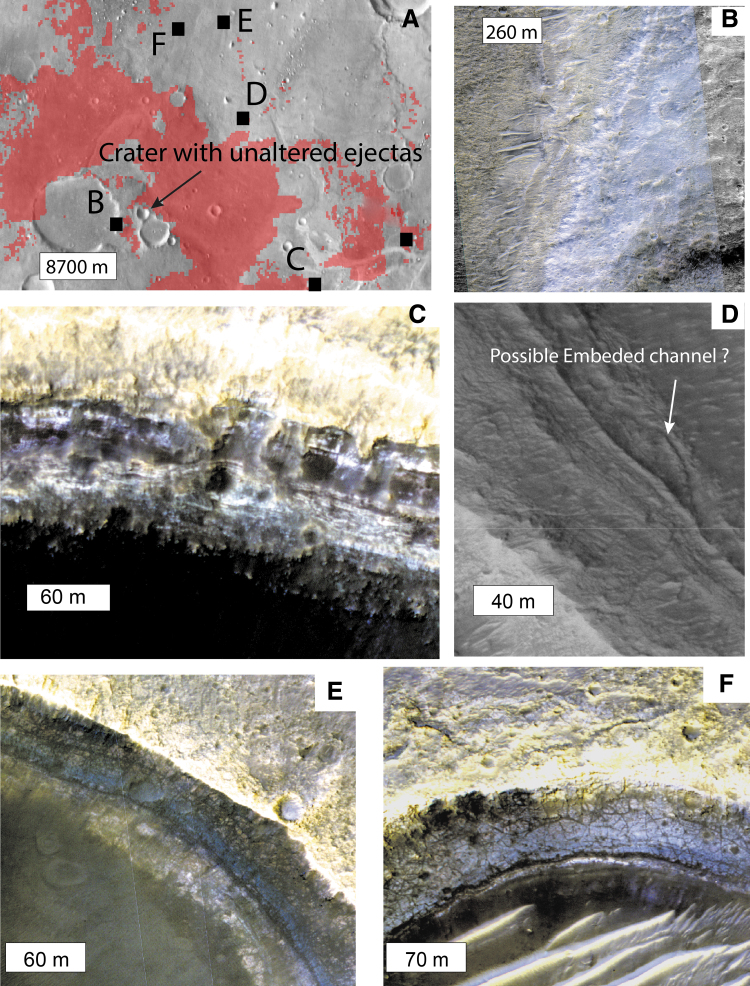
Layers of the Noachian clay-rich formation. **(A)** Location of the subfigures **B, C, D, E, F** and Fig. 7 and location of the 2.6 km crater with unaltered ejecta discussed in the article. The background is THEMIS daytime mosaic overlapped by the hydrated mineral mapping detailed in the work of Carter *et al.* (2016). **(B)** Rampart of an impact crater exposing ∼100 m of layers (HiRISE image ESP_044811_1985). **(C)** Layers exposed on the rampart of a 2 km impact crater. At least 50 m of layers are exposed here (HiRISE image ESP_043268_1980). **(D)** Possible cross-stratifications exposed in impact crater walls (HiRISE image PSP_003195_1985). **(E)** Layers exposed in the wall of a 1.3 km impact crater (HiRISE image ESP_051905_1990). **(F)** layers exposed in the wall of a 1.1 km impact crater (HiRISE image ESP_050705_1985). HiRISE, High Resolution Imaging Science Experiment; THEMIS, Thermal Emission Imaging System.

Bedrock regions with high clay abundance correlate spatially with geomorphic surfaces of specific morphology and appear to have consistent stratigraphic relationships with other terrain types in the region. This suggests that the clay-bearing bedrock represents a defined morphostratigraphic unit. Thanks to the near-complete coverage of HiRISE within the landing ellipses (Supplementary Fig. S1), the meter-scale characterization of the morphology of the unit is possible almost everywhere in the ellipse. The hydrous mineral signal in the spectral data always corresponds to widely exposed, light-toned, fractured material (Fig. 6). The fractures range from meters to decameters in spacing, are widespread, and seem to affect all the whole clay-rich layered formation. No clay-bearing material is observed without this fracture pattern.

**FIG. 7.**
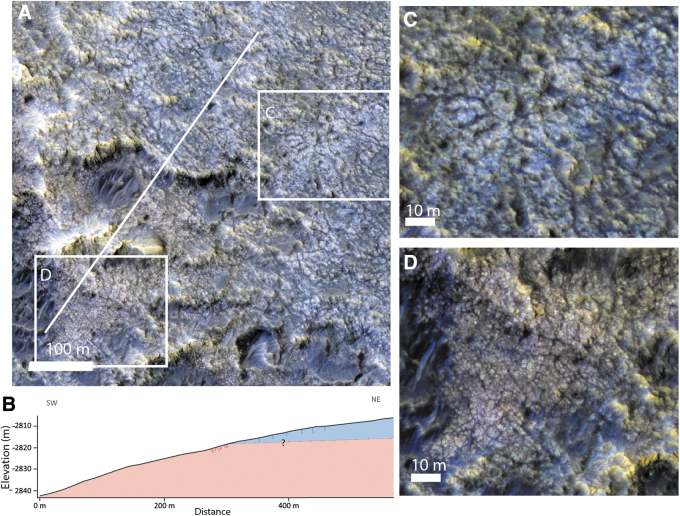
High-resolution morphologies and color variations of the fractured clay unit, as seen in HiRISE color data of ESP_045378_1980. **(A)** Closeup on the transition from upper and bluish unit and the reddish lower unit with the location of the topographic profile displayed in B and the two closeup **(C, D)**. **(B)** Topographic profile from CTX DTM and interpretative cross-section. **(C)** Closeup on the bluish upper unit with decametric fractures. **(D)** Closeup on the reddish lower unit with metric-scale fractures. CTX, Context Camera; DTM, Digital Terrain Model.

As shown in Fig. 6, the clay-bearing unit is layered, with thicknesses of individual layers ranging from 0.7 to 3 m as measured on HiRISE DTMs (Supplementary Fig. S2). The total thickness of the layered sequence is ∼100 m, based on the exposure observed in the inner wall of a 16 km degraded crater south of the landing ellipses (Fig. 6B). No clear nonlayered basement has been observed in the Oxia region, but there is some evidence that impact craters have penetrated beneath the clay-bearing unit and excavated unaltered material from below. In particular, some craters within the clay-bearing unit have partial remnant ejecta blankets that show no hydration signature (*e.g.*, Fig. 6A), suggesting that an unaltered (or less-altered) basement is being excavated by craters of this size. The crater shown in Fig. 6A is 2.66 km in diameter. Using a minimum excavation depth to diameter ratio of 10%, we can infer that unaltered or less-altered geological materials are present ∼260 m below the surface.

By analyzing HiRISE images, we identified at least two subunits (members) of the clay-bearing unit, which display different fracture size and distinct relative color in HiRISE. The first member, which always appears to be superposed by the second member, shows more closely spaced fractures, typically of meter scale, and has a reddish tone in HiRISE color products compared with the other member. The second member, always stratigraphically above the first, shows larger fracture patterns of decameter scale and a bluish tone in HiRISE color images. Both members contain clay-bearing material, according to spectral data. In topographically flat areas, such as the center part of the ellipses, the two members seem to be mixed at HiRISE scale, but some local slopes allow us to observe that the bluish member is lying on top of the reddish member (Fig. 7). The bluish member is as thick as 15 m where it is observed. The color difference in HiRISE data may be explained by different factors, including the surface roughness or the dust coverage, but may also be linked to compositional variations (McEwen *et al.*, 2007).

### 4.2. Fluvial-deltaic system and possible related units (Fd, Dt)

**FIG. 8.**
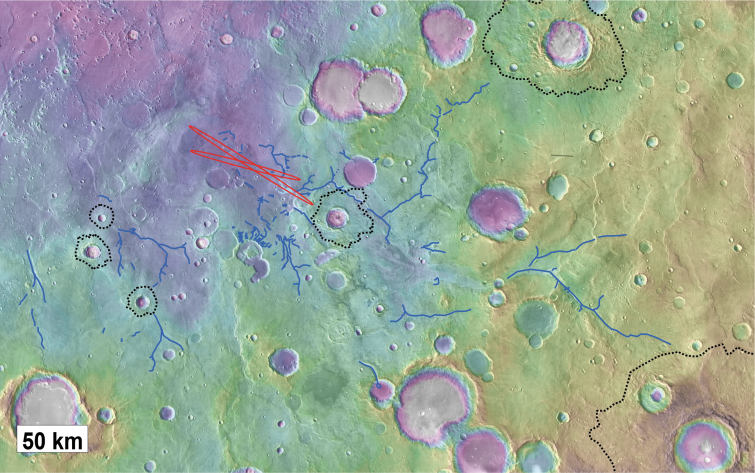
Catchment area and valley networks converging to Oxia Planum. The valley networks as depressions or as inverted channels are mapped as blue lines. The LPO and LPC ellipses of Oxia Planum are mapped in red. The impact craters with preserved ejectas are underlined as dotted dark lines. The valley network system is largely overlapped by these nondegraded craters.

**FIG. 9.**
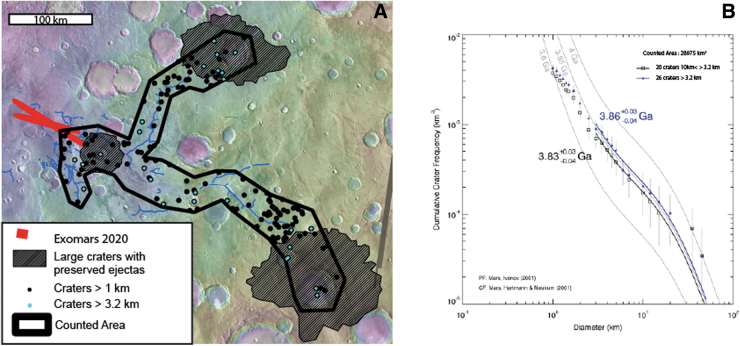
**(A)** Map of the craters identified as younger than Coogoon Valles fluvial system used in **(B)** for the age estimation. The three largest craters postdating the main fluvial activity are mapped as hatched with their ejecta. **(B)** Cumulative crater size distribution returning an age of 3.86 Ga with the Craterstats2 software (Michael *et al.*, 2012). The size distribution of craters >3.2 km (marked in blue) follows an isochron and was used to assess the age of the valley system. If we include all craters >3.2 km, the age returned is 3.86 Ga, while the age is 3.83 Ga if the six largest ones are excluded (as they are not perfectly aligned on the 3.86 isochron).

The ExoMars ellipses contain part of a major fluviodeltaic sedimentary system of the eastern margin of Chryse Planitia. The landing ellipses are located at the outlet of a 400 km-long fluvial bedrock-incised valley system, Coogoon Valles (Fig. 8). The drainage pattern appears to have few tributary branches (Fig. 8), but closer analysis shows that the original form of the valley system has been highly eroded and is superposed by large late Noachian and early Hesperian impact craters (dotted impact crater ejecta blankets are shown in Figs. 8 and 9), indicating that the valley network has been highly modified from its original state. We investigated the age of the fluvial landforms, analyzed their stratigraphic relationships with the other units, and quantified the crater retention age of the valley system. The timing of fluvial activity within the main catchment area of Coogoon has been determined by relative stratigraphy and crater counts (Fig. 9). This analysis indicates that fluvial erosion that created the valley system and led to the sediment fans described is younger than the layered clay-bearing unit and older than 3.86 Ga according to the size distribution of the craters >3.2 km (Fig. 9). Furthermore, Coogoon Valles has been partially infilled by material with high thermal inertia similar in texture and thermal inertia to the dark resistant unit described in the following sections, which prevents analysis of the channel floor within the valley system. This has been previously described in the work of Molina *et al.* (2017).

**FIG. 10.**
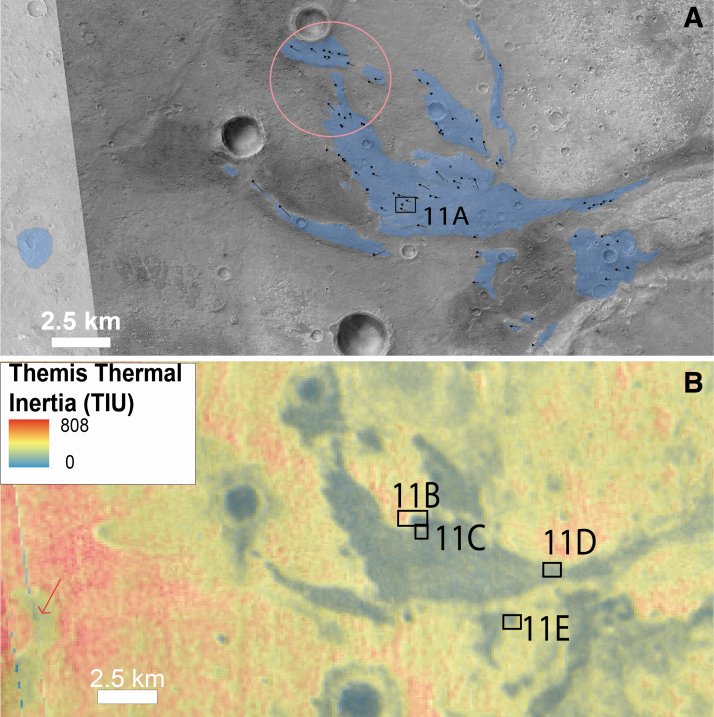
Overview of the delta fan: **(A)** CTX context where in blue the fan material has been mapped based on its thermal inertia signature. The arrow denotes the lineation observed on the top of the fan. The pink circle highlights the overlap of lineations of different orientations. The subfigure 11A is located. **(B)** THEMIS Thermal inertia map and location of the subfigures 11B–E. The red arrow points out the low thermal inertia fan at the outlet of another valley system than Coogoon Valles at similar elevation to the main delta fan.

**FIG. 11.**
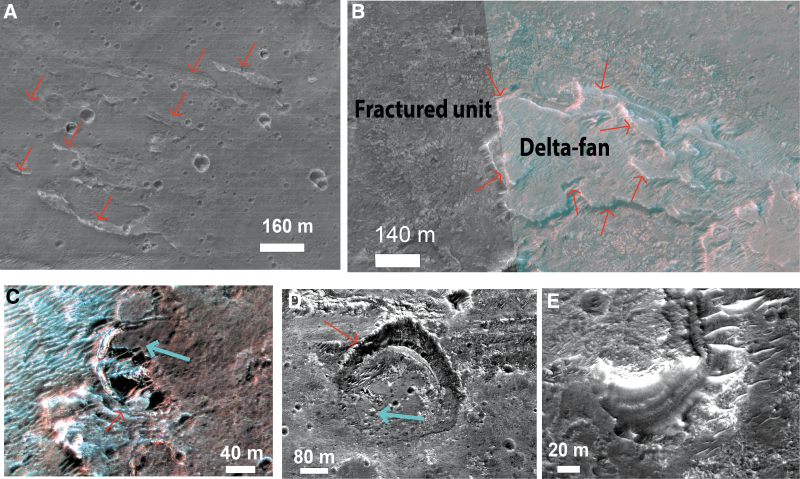
HiRISE views of the delta fan: **(A)** CTX closeup of lineations (part of CTX image G02_019084_1991_XI_19N024W). Red arrows point toward the lineations. **(B)** HiRISE closeup of a finger-type termination where we observe that the fan is lying on the top of the fractured unit (part of HiRISE ESP_019084_1980). The red arrows denote the retreating scarps. **(C, D)** HiRISE closeup of embedded impact craters within the fan in respective part of HiRISE ESP_019084_1980 for (C) and HiRISE ESP_039721_1980 for **(D)**. Red arrows denote the fan layers affected by the impact craters, attesting that the craters postdate a part of the fan activity and the blue arrows denote the fan material that filled the crater, attesting that the fan activity lasts after the craters emplacement. The direction of the blue arrow agrees with the putative flow sense. **(E)** HiRISE closeup of the layers exposed on the scarps of the fan (part of HiRISE ESP_039721_1980).

At the outlet of this drainage network, toward the eastern part of the landing ellipse, we observe a 10 km-long and 7 km-wide fan-shaped depositional sedimentary feature with divergent finger-like terminations that overlies the clay-bearing unit (Figs. 8, 10, and 11). The material comprising this deposit shows low thermal inertia (<100 TIU) compared with the surrounding clay-bearing unit, which has thermal inertia of 550–650 TIU (Fig. 10B). We used THEMIS night-time imagery to map the distribution of the fan deposit, which consequently appears darker in this data set than its surroundings. We estimate that it has an area of ∼25 km^2^ (Fig. 10A). The fan-shaped body is finely layered (Fig. 11E) and is ∼80 m thick in its thickest exposed section, as measured by using a CTX DTM. Many lineations are observed at the surface of the fan, many of which are overlapping and diverging (pink circle, Fig. 10A). These are interpreted as possibly the surface expression of channel infill sedimentary structures (Figs. 10A and 11A). This is comparable with similar fan structures observed in better preserved examples at the Hypanis delta on the other side of Chryse Planitia (Fawdon *et al.*, 2018) and at the Jezero sedimentary fan in Nili fossae region (Fassett *et al.*, 2007; Goudge *et al.*, 2017).

The fan-shaped sedimentary body could have three possible interpretations: (1) a subaerial fluvial fan deposit, (2) a deltaic fan deposit, or (3) a submarine fan deposit. Here, we favor a deltaic fan interpretation based on the observation that the fan deposit comprises a set of overlapping elongated finger-like terminations that we interpret as possible individual delta lobes formed at the mouth of distributary channels. Similar features are observed on other sedimentary fans interpreted as exhumed ancient delta fans on Mars: Eberswalde delta (Pondrelli *et al.*, 2008), Jezero delta (Goudge *et al.*, 2017), or the Hypanis delta system (Fawdon *et al.*, 2018). On Earth, there is no example of preserved ancient delta fans exposed well enough at the surface to be investigated from orbital data, so the analogy can only be made with active deltaic systems. The divergent finger terminations we have observed in the Oxia fan may represent inverted channel bifurcation, comparable with the divergence of distributary channels observed at the top of active terrestrial deltas (Chamberlain *et al.*, 2018). We note that the fan deposit is an erosional remnant, and its preserved morphology does not represent the original morphology. Nevertheless, the presence of multiple finger-like projections and its presence at the margin of an extensive basin may suggest formation at the margin of a standing body of water. This is a hypothesis that will be tested by *in situ* observations by the Rosalind Franklin rover. The fan is currently undergoing erosion, as attested by the retreating scarp exposing possible remnant inverted channels of a delta system (Fig. 11B). On these scarps, hydrated silica signatures are detected in CRISM data (Carter *et al.*, 2016). In fact, the rocks that compose the sediment-fan layers are unique within the study area, in that they are enriched in hydrated silica. This points to water–rock interactions with different conditions than those existed during the formation of the clay-rich unit (Carter *et al.*, 2016).

**FIG. 12.**
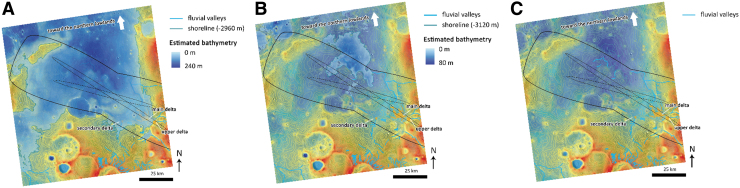
Main water levels recorded in Oxia Planum (background is MOLA topography): **(A)** the different delta fans around −2960 m of elevation denote first water level. The upper delta implies a higher water level at around −2940 m, while the secondary delta implies a lower water level at around −3010 m. In such case, the bathymetry would be of the order of 240 m for the deepest point of the basin, and the standing body of water would be open to the northern plains according to the current topography. **(B)** A second main water level is recorded at the outlet of the valleys downstream the delta fans at around −3120 m. Some valleys imply an even lower water level, indicating a restricted standing body of water of 80 m for the deepest part. According to the current topography, the basin would be enclosed and separated from Chryse Planitia. **(C)** Current dry conditions.

If the fan-shaped sedimentary body is an exhumed ancient delta deposit, this argues for the former presence of a standing body of water that likely covered almost the entire landing ellipse. Indeed, the elevations of most of the landing ellipse are lower than the delta fan and the putative body of water (Fig. 12). We note that in the present-day topography, the northern part of Oxia Planum is not delimited by any significant positive relief. Therefore, it is possible that the standing body of water, required by the presence of a delta fan, was open to the northern lowlands (Fig. 12).

Another possible fan deposit is also observed at the same elevation as the main delta fan and at the outlet of another valley system, immediately southwest of the main delta fan (Fig. 10A). Below the main delta fan, shallow valleys are observed, arguing for another stage of fluvial activity, possibly correlated with a lower level of the standing body of water (Figs. 8 and 12). There is no obvious evidence for this lower level of water before or after the water level related to the main delta fan. However, as we know that Mars dried out with time (*e.g.*, Quantin-Nataf *et al.*, 2019), we assume in the discussion section on the geological evolution of Oxia Planum that this lower water level more likely postdated the upper level of water comparable with sediment-fan evolution at Hypanis Vallis delta system (Fawdon *et al.*, 2018).

Several impact craters are interbedded within the delta-fan stratigraphy (Fig. 11D, E), showing that the formation of the delta deposit continued over an extended time, allowing multiple impact craters to accumulate. The craters excavated existing fan materials but were later buried by additional fan sediment. Two of the largest interbedded craters have diameters between 250 and 320 m (*e.g.*, Fig. 11D). Using crater age models, we can assess the order of magnitude of the lapse of time required between two impact craters of that size at different periods of martian time. If we use a Hartmann size distribution (Hartmann, 2005) that is well defined for small size crater and the impact rate from Neukum *et al.* (2001), we estimate that at 4 Ga, the frequency of impact craters from 250 to 320 m over a surface of 52 km^2^ is ∼9.7 Ma year. At 3.7 Ga, crater of such size would occur every ∼76 Ma and at 3.5 Ga, they would typically occur every ∼292 Ma.

In summary, the observation of more than one interbedded crater of 250–300 m diameter implies that the emplacement of the delta fan had a duration of tens of millions to several tens of millions of years, depending on when it is formed. Such a duration is long compared with terrestrial delta formation. For instance, terrestrial kilometer-scale lacustrine deltas can be formed in less than a thousand years (Stevens and Robinson, 2007). A more credible alternative is that there were hiatuses or intermittency in the deposition of a similar length of time.

In terms of sediment volume balance, it is unlikely that the delta fan is the only deposit of the eroded sediments transported from the upstream valley network. Given the size of the upstream valley networks (<400 km long) and the current depth of the valleys (>200 m for the main stem of Coogoon Valles and >50 m for the upstream valleys), the eroded volume in the watershed is about two orders of magnitude larger than the volume of the delta fan. In addition, if a standing body of water was present, we expect that other deposits formed downstream of the current remnant delta, that is, in the lowest part of the landing site uncertainty ellipse. On Earth, deltaic deposits formed by fluvial sediment supply are associated with a more widespread depositional environment where distal mudstone or sandstones form at the front of the deltas (Bhattacharya, 2007). Such deposits, if ever formed, might be preserved as outliers of layered material overlying the clay-bearing units within the landing site study area. From orbit, no obvious deposits clearly linked to the main delta fan have been observed so far.

### 4.3. Remnant rounded buttes (Rb)

**FIG. 13.**
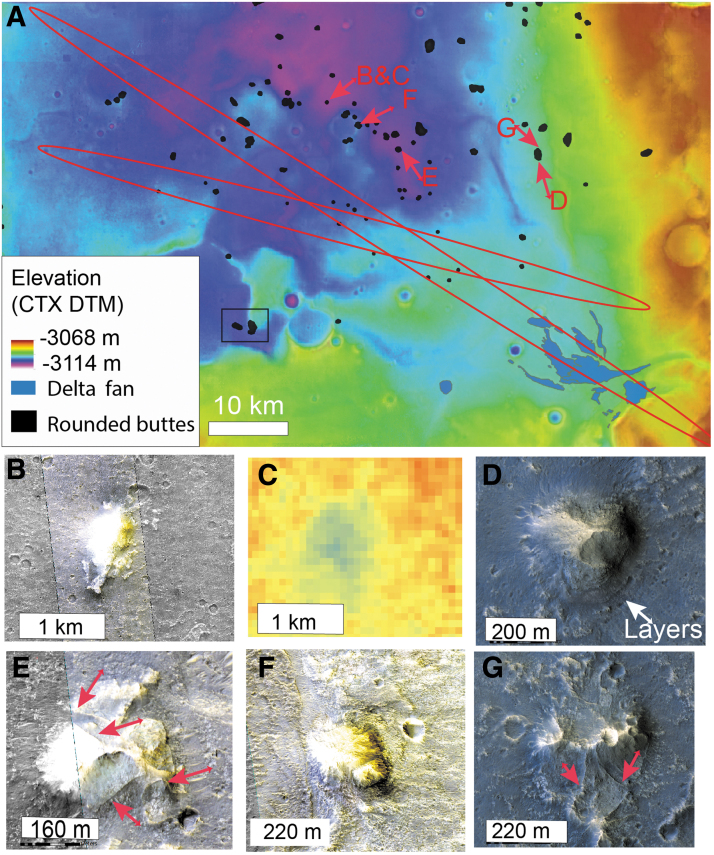
Rounded buttes distribution. **(A)** Location of rounded buttes mapped in black. The red ellipses are LPO and LPC landing ellipses. The background is a mosaic of CTX DTM. The square denotes the location of Figure 14. **(B)** HiRISE closeup of a rounded butte (ESP_051905_1990). **(C)** THEMIS thermal inertia map of the rounded butte displayed in **(B)**. The range of thermal inertia is from 0 to 800 TIU. The rounded butte has a clear low thermal inertia signature that cannot be explained by recent loose material since the lowest thermal inertia is observed at the top of the butte. **(D)** HiRISE closeup of a rounded butte showing an impressive exposure of layers (ESP_037070_1985). **(E)** HiRISE closeup of a rounded butte displaying inverted ridges marked as red arrows (ESP_044679_1985). **(F)** HiRISE closeup of a rounded butte lying on a crater rim (ESP_51206_1985). **(G)** HiRISE closeup of a rounded butte displaying inverted ridges marked as red arrow (ESP_037070_1985).

Scattered rounded buttes of hundreds of meters to a few kilometers in diameter are found located in the vicinity of the delta-fan deposit and down slope into Chryse Planitia. They are more common approaching Chryse Planitia, but some occur within the ExoMars ellipses (Fig. 13). They are easily identified on thermal inertia maps as they show a clear low thermal inertia signal (Fig. 13C). As the low thermal inertia signal includes the summit of these buttes (Fig. 13B), it is likely the low thermal inertia is not due to loose material on the flanks, but rather an intrinsic signature of the bulk material of these buttes. In HiRISE images, we observe that these buttes are layered and contain narrow (< meter-scale) positive relief, curvilinear ridges. These lineations appear indurated, highlighted by differential erosion (Fig. 13D, E, G).

**FIG. 14.**
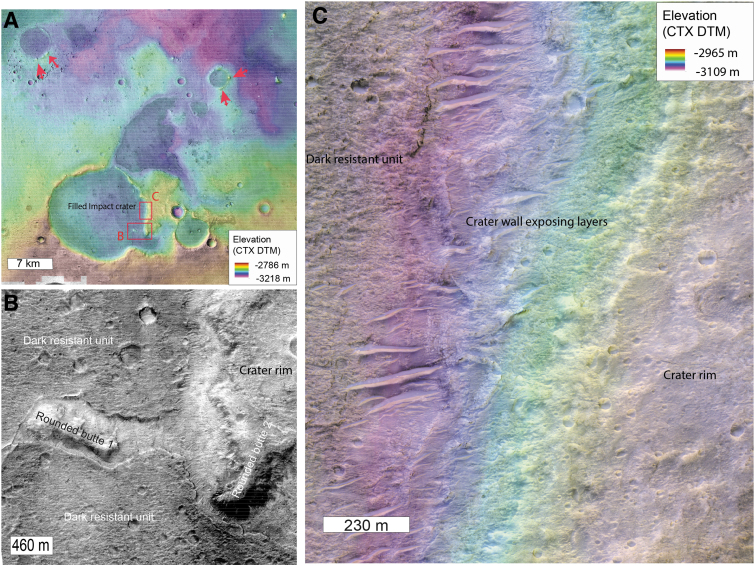
Rounded buttes stratigraphy. **(A)** Global topographic view of a 15 km crater filled by the dark resistant unit. The background is a mosaic of CTX images with CTX DTM topography displayed in transparency. The red arrows locate the rounded buttes observed on the rim of impact craters. The two red squares denote the location of the subfigure **(B, C)**. **(B)** HiRISE closeup of the rounded buttes linked to the 15 km crater (ESP_044811_1985). The rounded butte 1 is on the floor of the impact crater, while the rounded butte 2 is on the rim of the crater. They both are surrounded by the dark resistant unit, suggesting that the dark resistant unit embedded the rounded buttes. This observation implies that the rounded buttes predate the dark resistant unit's emplacement and where already as remnant buttes by that time. **(C)** HiRISE closeup of the rim of the 15 km crater (ESP_44811_1985). CTX DTM topography is superimposed in transparency. We note the light-toned layers exposed in the inner wall of the impact crater.

The buttes overlie the clay-bearing unit, suggesting that they are younger, and are possibly erosional remnants of slightly younger, overlying sediments. Unusually, the buttes are sometimes located on the rim of eroded impact craters, set into the clay-bearing unit (see red arrows on Fig. 14A or Fig. 13F). In the center of Oxia Planum, a 15 km crater exposes light-toned layers typical of the clay-bearing unit in its walls, demonstrating that the crater was emplaced within the clay-rich layers (Fig. 14C). This large crater is filled by the dark resistant unit described later in the article. Rounded buttes are observed on the rim of the crater but also as local highs within the dark resistant unit on the floor of the crater (Fig. 14B). The dark resistant unit surrounds the buttes, suggesting that it embayed the buttes (Fig. 14B). This implies that the buttes predate the emplacement of the dark resistant unit. The observation of buttes on the rim of impact craters emplaced within the clay-bearing unit suggests that the buttes formed long enough after the clay-bearing layered formation for large craters to form. Two scenarios can be proposed for their origin. Either they were formed as isolated buttes with a denser occurrence toward Chryse Planitia, or they are erosional remnants of a pre-existing continuous or semicontinuous deposit that is now better preserved toward Chryse Planitia. No obvious cap rock has however been observed on the top of the buttes to explain their ubiquitous preservation.

Similar rounded buttes are observed elsewhere in the circum-Chryse region: in the region of the Hypanis delta, where they similarly occur more frequently toward Chryse basin and are also in some places located on the rim of impact craters (Fawdon *et al.*, 2018). Both the deltaic deposits in Oxia Planum and Hypanis region and the rounded buttes are made of a similar low thermal inertia material. No clear signature of hydrated minerals related to the buttes has been found so far, but there is no targeted CRISM observation over large enough examples to allow for mineralogical identification at CRISM scale.

The fact that both the deltaic deposits and the rounded buttes are made of similar low thermal inertia material, their layered morphology and similarity the rounded buttes at the outlet of channels in the circum-Chryse region, and their occurrence at a lower elevation than the outlet of main channels around Chryse favor the hypothesis of these being remnants of a wider sedimentary deposit, possibly linked with a standing body of water in Chryse Planitia (Fig. 12). Regardless of the origin of the buttes, the fact that the dark resistant unit embays the buttes means that by the time of the dark resistant unit's emplacement, the buttes were already in their current eroded form. In the scenario that these rounded buttes once belonged to a widespread deposit, significant amounts of erosion would have occurred in this region after the emplacement of the clay-bearing unit and before the emplacement of the dark resistant unit.

### 4.4. The mantling unit

**FIG. 15.**
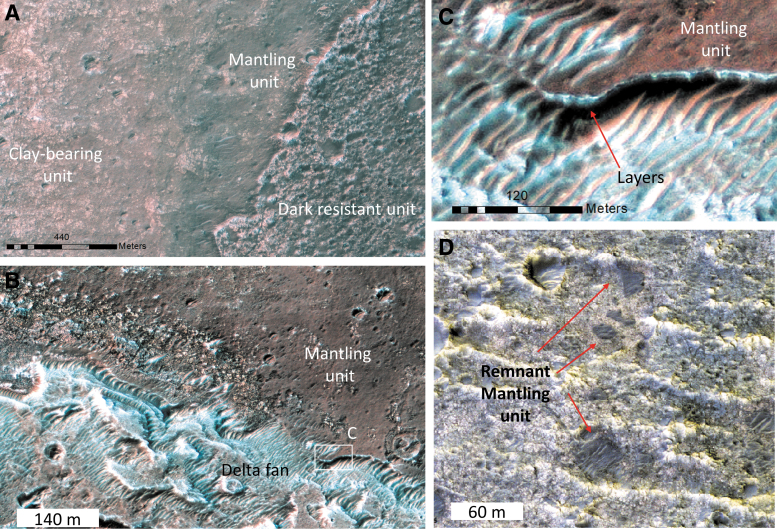
The mantling unit. **(A)** HiRISE view of the transition between the dark resistant unit, the mantling unit, and the clay-bearing unit (ESP_019084_1980). The mantling unit is covering the clay-bearing unit but has never been observed at the top of the dark resistant unit. **(B)** HiRISE closeup of the mantling unit right at the feet of the delta fan (ESP_019084_1980). The mantling unit is often observed around and downstream the main delta fan. **(C)** HiRISE closeup of the flank exposure of the mantling unit (ESP_019084_1980). Layers seem to be present. The unit is indurated and ∼5 m thick as measured here on a HiRISE DTM. **(D)** HiRISE closeup of the mantling unit in the center part of the landing ellipse where the clay-rich unit is more exposed. There, the mantling unit is less extensive and is only observed as remnants in local lows such as impact craters or as inverted impact craters.

We have identified a thin capping unit we named “the mantling unit.” In locations where no dark resistant unit is observed and no clay signature is present, we observe that the fractured layered clay-rich unit is overlain by a thin, mid-toned, mantling unit (Fig. 15) that drapes the underlying relief. This unit is indurated and layered, with a measured thickness of only ∼5 m in the thickest part, north of the delta fan. The mantling unit is widespread downstream of the remnant delta where many transverse aeolian ridges (*e.g.*, Balme *et al.*, 2008) are observed. However, the unit is scarp forming, and retains small impact craters so it is lithified, and not a recent blanket of loose materials. Away from the delta fan in the other parts of the ellipse, the clay-rich unit is more exposed, but the mantling unit is still observed as remnants, which appear to be inverted crater fills (Fig. 15D). We infer, therefore, that this unit was once more extensive but has since been eroded, more so in the central part of the ellipse than near the delta (or the unit was initially thicker near the delta). The mantling unit is found to systematically superpose the clay-bearing unit and seems also to superpose the flanks of the delta fan. It has not been observed to superpose the dark resistant unit (Fig. 15A). It may represent an erosional product either predating the dark resistant unit or a more recent, probably active, erosional product. If this mantling unit is a recent erosional product, the fact that the dark resistant unit does not contain small outliers of this mantling unit implies that the resistant unit is not affected by the process.

### 4.5. Dark resistant unit (Adru)

**FIG. 16.**
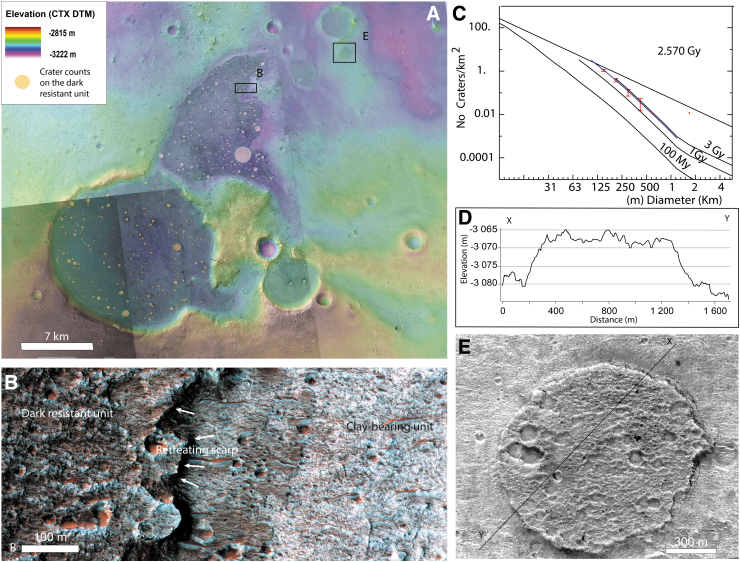
The dark resistant unit: **(A)** CTX image mosaic superimposed by CTX DTM mosaic showing that the dark resistant unit is in topographic lows. Black squares locate the subfigure **(B, E)**. **(B)** HiRISE closeup of the border of the dark resistant unit where the retreating scarp allows the subjacent clay-bearing unit to be freshly exposed (PSP_009735_1985), **(C)** Crater counts corresponding to the pinkish craters mapped in **(A)**. Incremental crater size distribution with an age model displayed corresponding to Hartmann (2005) isochrones and Ivanov (2001) impact rate model. The crater size distribution observed follows the isochrons slope with best age model of 2.57 Ga. The counted area is 83.75 km^2^ where 413 craters have been mapped. **(D)** Topographic profile from X and Y points displayed in **(E)**. Elevation has been extracted from CTX DTM mosaic. The dark resistant unit is here ∼15 m thick. **(E)** HiRISE closeup of an impact crater filled by the dark resistant capping unit as inverse morphology (ESP_051206_1985).

The dark resistant unit is defined as a dark-toned unit resistant to erosion. We have identified examples of a dark resistant unit everywhere in the ellipse; as large exposures in local topographic lows such as impact craters, ancient valleys, but also as small mesas (Figs. 4 and 16). It is easy to identify the unit based on its dark, rugged, and massive appearance, because it is stratigraphically on top of all other units described previously. At high resolution, the surface of the unit contains many impact craters and is comprised of a material with good crater-retaining properties. The unit is ∼20 m thick and shows the spectral features of mafic minerals (Carter *et al.*, 2016). No layering is observed in the exposed flanks and the surface of the dark resistant unit, and it displays many meter-scale boulders, significantly more than the clay-rich unit (Pajola *et al.*, 2017). At small scale, this unit is observed as inverted craters or inverted channels scattered across the study region. Inverted morphologies are observed when topographic lows are filled by material more resistant to erosion than the surrounding material. Then, differential erosion removes the surrounding material. Recognition of inverted morphological features in Oxia Planum suggests that the region has undergone significant erosion.

We performed crater counts on the largest exposures of the dark resistant unit and returned an Amazonian age of 2.6 Ga, based on the size–frequency distribution of 100–500 m craters. The age is constrained by different crater size bins following the isochrons but, as the counted area is small (83 km^2^), and we only have statistics for 100–500 m craters, we cannot rule out an older age (Fig. 16). The dark resistant unit has probably “captured” the Oxia landscapes at their erosional stage ∼2.6 Ga ago, preserving them beneath it. Further erosion of the dark resistant unit has now revealed the older strata.

Two origins can be proposed for the characteristics and the context of the dark resistant unit: a sedimentary origin and a volcanic origin. As many of the remnant morphologies are either in local topographic lows or as inverted local topographic lows (impact crater or valleys), the unit may have been deposited as water-related sediments: as fluvial deposits within the valleys and as lacustrine deposits within the craters. For instance, a previous study in the region interpreted the same unit within craters as possibly deposited in a stable water body that was contained by the crater topography (Molina *et al.*, 2017). This may be a relevant hypothesis for large impact craters and valleys, but it is difficult to envisage for a small isolated crater lake <100s meters in diameter, and without any fluvial dissection on the rim of the crater. Moreover, no hydrated minerals have been found so far, and instead, mafic compositions are inferred from the spectral signature of the unit.

In case of a sedimentary origin, the mineralogy as seen from orbit would imply transport of mafic-rich material by limited, short-lived, water-related processes to explain the absence of alteration minerals, especially compared with the surrounding material. A caveat about the absence of hydrated mineral detection may be that it is an observational bias. If we compare this site with the sedimentary site of Gale Crater visited by the Mars Science Laboratory mission, no hydrated minerals were detected from orbit at the Yellowknife bay site, yet were reported by the rover *in situ* (Vaniman *et al.*, 2014). Also in Gale Crater, there is a large part of detrital igneous phases in the fluviodeltaic deposits (Treiman *et al.*, 2016; Bedford *et al.*, 2019, 2020). The hypothesis of fluviolacustrine deposits cannot therefore be completely ruled out by the absence of hydrated minerals alone. Aeolian sandstones are also a possible interpretation, but aeolian deposits would be expected to form on slopes and not within topographic lows only. Such a hypothesis would require an extensive wide aeolian deposit eroded and preserved only in local topographic lows. There is no evidence of remnants of such extended aeolian mafic-rich deposits elsewhere in the area.

The mafic composition, the massive appearance, and the resistance to erosion are all features that better support the hypothesis of a volcanic origin (explosive deposits or lava flows). As the inverted craters and valleys filled by the dark resistant unit are scattered across the entire region of Oxia Planum, the unit may once have been more extensive or even covered the entire region. This hypothesis would imply that the unit has been preserved from erosion in local topographic lows rather than initial emplacement only in topographic lows. Also, in case of effusive volcanism, we expect the deposits to be thicker in topographic depressions and consequently to be resistant longer to erosion in those locations.

If this unit was emplaced as lava flows, several hypotheses for their origin can be proposed. First, the source of the lavas could have been local volcanoes. For example, a topographic dome south of the ellipse contains an irregular, noncircular depression and has a convex shape to its flanks (Supplementary Fig. S2). The dome has a convex shape (Supplementary Fig. S2), similarly to the ones observed elsewhere in Arabia Terra (Michalski and Bleacher, 2013), which are interpreted as volcanic domes. We note that the flank of this dome shows no hydrated mineral signature, which suggests that at least the latest stages of flank formation postdate deposition of the clay-bearing unit (Fig. 3). These domes are plausibly interpreted as volcanic domes that could have been the source of the lava flow that extended into the ellipse.

Second, as Oxia Planum is located near the martian dichotomy, it is also at the edge of the volcanic material that forms the fill of the martian northern lowlands (Tanaka *et al.*, 2014). The dark resistant unit could be a thin, remnant edge of the northern lowlands volcanic infill. Some authors suspect that rocks emplaced by water-related processes are buried below the volcanic infilling of northern lowlands (Carter *et al.*, 2010; Pan *et al.*, 2017), so if this was the case, Oxia Planum is an important location where rocks older than the volcanic infill of northern lowlands are exposed and reachable by rover investigation. These two hypotheses would differ in the sense of flow that would be northward in case of a local source from the volcanic dome and southward from Chryse in case of the global lowlands infilling. So far, no clear evidence either of flow or sense of flow has been observed. Hence, alternative hypotheses such as resistant volcanosedimentary deposits or sandstones are still possible scenarios.

### 4.6. Erosional history of Oxia Planum

**FIG. 17.**
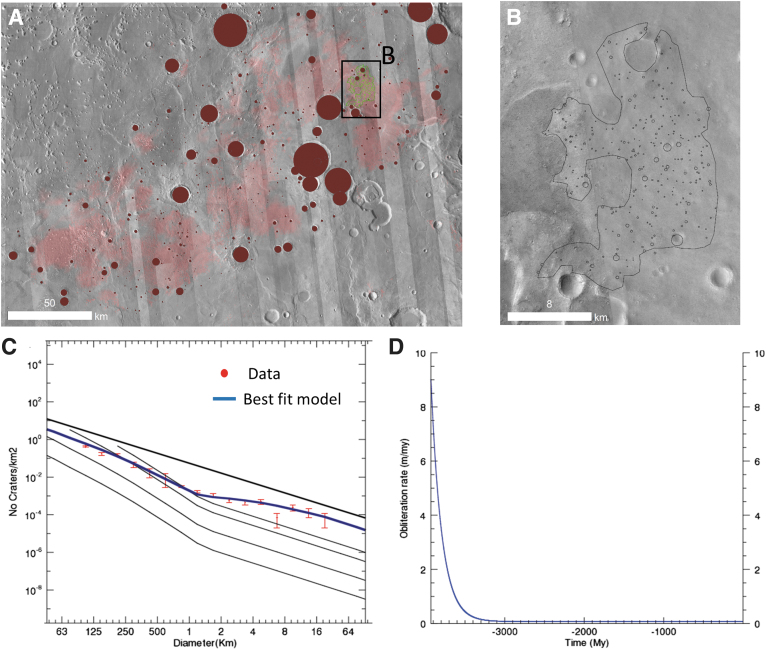
Crater obliteration history. **(A)** global crater count on the clay-bearing unit. Light red corresponds to the hydrated mineral mapping from the work of Carter *et al.* (2016). The craters >1 km (in red) have been assessed over the entire Oxia Planum area at THEMIS image scale. Four hundred two craters have been mapped over an area of 28,626 km^2^. **(B)** Crater counts done at higher spatial resolution on CTX images on a part of well-exposed clay-bearing unit. Two hundred fifty-six craters <1 km have been mapped on a surface of 216 km^2^. **(C)** In red is the entire crater size distribution of crater from 100 m to 30 km of diameter from the crater count presented in A and B. In blue is the modeled crater size distribution according to the crater obliteration model presented in D that best fits the data. The methodology of the modeling and best fitting is presented in the work of Quantin-Nataf *et al.* (2019). **(D)** The crater obliteration evolution of the best fit model of crater size distribution. Like Mars globally, Oxia Planum has undergone a decrease of crater obliteration rate with time from a rate as large as 8 m/Ma in Noachian. The total amount of crater obliteration is in the order of 900 m. It is distributed as 650 m between 4 and 3 Ga and 240 m over the last 3 Ga.

The entire landing ellipse shows evidence for extensive erosion. First, none of the small crater populations in any of the geological units follow the crater size distribution of the expected production function, indicating continuous crater removal (or burial) processes (Quantin-Nataf *et al.*, 2019). In addition, many inverted morphologies, such as inverted impact craters (Fig. 16E) or inverted channels or valleys (Fig. 8), are observed. In an attempt to obtain quantitative constraints, we used the model from a previous study (Quantin-Nataf *et al.*, 2019) to assess the crater obliteration rate through time from the crater size distribution. We obtained impact crater size–frequency data at two different scales on the clay-bearing unit. We measured the crater size distribution of craters >1 km over the entire clay-bearing unit in Oxia Planum and the size distribution of craters >63 m (using CTX data) on a smaller portion of Oxia clay exposure inside the landing ellipse (Fig. 17). This gives us the complete crater size distribution from 63 m to 16 km diameter.

We then applied the method described in the work of Quantin-Nataf *et al.* (2019) to calculate the obliteration rate evolution that best fits the observed crater size distribution. The model uses an exponential decrease of crater obliteration rate with time, with two free parameters returned by our best fit approach: the initial and current rates of obliteration. It has been demonstrated that Mars at a global scale has undergone an exponential decrease of crater obliteration rate with time from Noachian until now (Quantin-Nataf *et al.*, 2019). The best model of the crater obliteration rate for Oxia Planum perfectly reproduced the observed crater size distribution (Fig. 17). A crater obliteration rate as high as 8 m/Ma is required during the Noachian to reproduce the lack of large craters and then an exponential decrease until values of ∼8 cm/Ma today. Note that during the Hesperian, the crater obliteration rate was still as high as ∼2 m/Ma. By using the integral of the curve, a total of ∼900 m of relief would have been erased over the last 4 Ga. These 900 m are distributed as 650 m during the first Ga and 250 m over the last 3 Ga. These crater obliteration rates include the removal of crater shape by either infilling or erosion and demonstrate the vigor of the sedimentary cycle (Quantin-Nataf *et al.*, 2019). As the current morphology of Oxia Planum records a history of erosion, we interpret the Amazonian crater obliteration to be due to erosional, rather than depositional, processes and provide ∼250 m of erosion over the last 3 Ga. For Noachian and Hesperian times, we have no clear morphological evidence of erosion versus deposition, but we know from the deposition of the clay-rich unit first and then from the fluviodeltaic system, that the sedimentary cycle was active.

## 5. Discussion

### 5.1. Reconstruction of the geological evolution of Oxia Planum

**FIG. 18.**
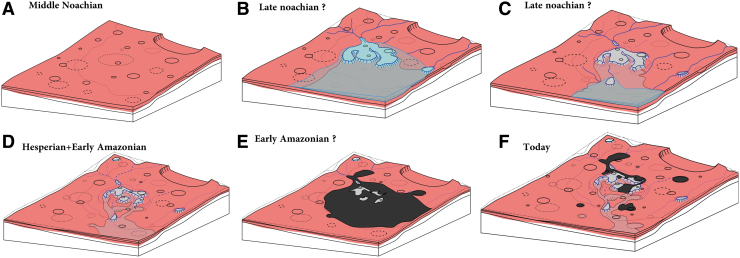
Proposed scenario of the evolution of Oxia Planum. **(A)** In middle Noachian, the layered clay-rich unit forms. **(B)** Much later than the clay-rich layered formation but still during Noachian, a fluviodeltaic system occurs depositing the currently observed main delta fan. Extended deposits corresponding to the remnant buttes observed today may have formed during this period. **(C)** Later the water level decreases allowing erosion of the main delta fan and eroding valleys downstream the main delta fan as well as all other deposits. **(D)** During Hesperian time and early Amazonian times, a large amount of erosion occurred leading to remnant rounded buttes. **(E)** A widespread unit emplaced (the dark resistant unit) possibly by volcanism, like lava flow process. **(F)** After the early Amazonian, erosion continues leading to the erosion of the dark resistant unit allowing for the exposure of the eroded landscape at their stage of early Amazonian times.

In summary, the geological history of the region can be reconstructed as follows (Fig. 18): (1) the crustal dichotomy was formed before the formation and alteration of the geological units observed at Oxia Planum. (2) During the mid-Noachian or earlier, the layered clay-bearing formation was deposited and aqueously altered (Fig. 18A), forming extensive clay-bearing sedimentary rocks. From our orbital investigation, it is impossible to decipher the age of the alteration itself. (3) Later in the Noachian, the large catchment and the related delta fan formed (Fig. 18B). Evidence of several levels of water is observed. In Fig. 18, we assume a decrease of the water level with time, but no clear stratigraphic relationships attest to the timing of these different water levels (Fig. 18C). (4) During Hesperian times, a significant amount of erosion should have occurred to shape the rounded buttes, reworking the bulk of the earlier deposits and possibly depositing the mantling unit (Fig. 18D). (5) The dark resistant unit was deposited last, possibly as extrusive volcanic materials during the early Amazonian (Fig. 18E). (6) During the Amazonian (since ∼2.9 Ga), continued long-term wind erosion likely affected the dark resistant unit, exposing the subjacent units (Fig. 18F).

### 5.2. Origin of the layered and fractured clay-bearing unit

The crater-count age and stratigraphic relationships with the global martian geological units (Tanaka *et al.*, 2014) suggest that the clay-bearing unit is mid-Noachian in age. The unit appears to drape paleotopography at a regional scale (Fig. 3), which could be explained by several processes, including air-fall deposits (*e.g.*, ash-fall) or sedimentary deposits covering a pre-existing topography. The draping nature of the unit is not in agreement with an origin by extrusive volcanism as one would expect lava flows to fill the lower regions. Alteration into clays may have occurred before deposition (detrital clays), during deposition or after (*e.g.*, by pedogenesis). As resolved by HiRISE images, the clay-rich unit is finely layered, with layers typically <1 m. This is compatible with sedimentary deposits formed by the deposition from liquid water in either palustrine, lacustrine, or marine contexts. This is also compatible with volcaniclastic sediments (*e.g.*, ashes) altered by meteoritic water. However, we cannot rule out a thin lava flow stack that experienced alteration after emplacement. The nature of the clay minerals is not consistent with hydrothermal alteration or metamorphism (Carter *et al.*, 2016), but meteoric-sourced water alteration of lava flow or ash deposits remains a possible scenario. The clay-bearing, light-toned layered material observed in Oxia Planum seems to be more widespread along the border of Chryse Planitia, including the west side of Chryse basin or Mawrth Vallis region (Carter *et al.*, 2016). Due to the ancient age of the deposits, we must keep in mind that the geological context of these events as observable from orbit may have disappeared, obscuring the origin of the layers (volcanism or sedimentary deposits).

The clay-bearing unit is widely fractured. The fracturing of sedimentary rocks is often observed due to the changes in the stress regime during the burial and unloading of sediments. Two main processes can be activated: (1) hydraulic fracturing that occurred during the burial of hydrated sediments due to the increase of pore-fluid pressure, or (2) in case of an underlying ductile layer, the deformation of the ductile layers leading to the fracturing of more resistant strata (Caswell and Milliken, 2017). Both processes require pre-existing burial of the fractured material. In Gale crater, fracturing has been observed within the fluviolacustrine sedimentary rocks of both the Yellowknife Bay formation (Bradbury group) and the Murray formation (Mt Sharp group) (Caswell and Milliken, 2017). They have been interpreted by some authors to be the result of hydraulic fracturing (Caswell and Milliken, 2017). Models predict that such hydraulic fracturing would start on Mars from ∼1 km of burial (Caswell and Milliken, 2017). The mineralogy excludes hydrothermal or low-grade metamorphic processes linked with deep burial, but a limited burial of ∼1 km is consistent with the observed mineralogy. If the Oxia layered clays were once buried by ∼1 km of sediments, this would imply that at least this depth of erosion has occurred to expose the fractured layered clays. This is consistent with the results obtained by crater size distribution analysis presented in section “The Geology of the Oxia Planum ExoMars Rover Landing Site.”

Both Oxia Planum and Mawrth Vallis are below the water level of some putative ocean shorelines in Arabia Terra (*e.g.*, Carr, 2003 or Citron *et al.*, 2018). The nature of the clay minerals is not inconsistent with marine deposits (Carter *et al.*, 2016), so sediments from the primitive martian ocean will be a hypothesis to test when the ExoMars rover is on the ground.

If we assume a marine or lacustrine sedimentary origin for the clay-bearing unit, what can we learn from terrestrial sedimentary deposits? Terrestrial global-scale marine sedimentation rate can be estimated from the ratio of the thickness of the sediments over the oceanic crust (Divins, 2003) and the age of the oceanic crust (Piñero *et al.*, 2013). On Earth, these sedimentation rates range from 10^−2^ to 10^3^ cm/ka. The highest values are observed on the continental margin, while the deepest parts of the oceans have the lowest rates (Piñero *et al.*, 2013). We have no constraint on what the chemistry of an ancient ocean on Mars might have been, and so whether direct comparison between Earth and Mars can be made is debatable, but we could use these extremes of sedimentation rates to calculate how long an ocean on Mars would have had to remain liquid to produce the ∼100 m of sediments observed at Oxia Planum. Such a thickness of marine sediments would require from 10 ka (continental margin and environments of active erosion) to 1 Ga (deep ocean-like environments). So, if the clay-bearing units are sediments deposited in a standing body of water, the ∼100 m-thick sedimentary record either demonstrates a short-lived ocean under environment of active erosion, a longer lived ocean under low erosion environment, a partial record of the ocean-related sedimentary activity or episodic depositional activity. A similar discussion can be made for a palustrine environment. The main point here is that ∼100 m of sedimentation could have occurred in a fairly short period, or by longer intermittent or low rate processes.

Since the deposits overlap the current topography of the margin of Chryse Planitia, the deposition of the layers postdates the formation of the martian dichotomy, regardless of its origin and the formation of Chryse Planitia, which probably originated from a giant impact (>1000 km) >4 Gy ago (Pan *et al.*, 2019). The ExoMars landing ellipses lie in a possible remnant circular depression (Fig. 3) that may have been a large impact crater (>100 km) but which is now open toward Chryse Planitia. Assuming that this 100 km depression was originally an impact crater, the layers that overlap and postdate the current topography were deposited after its formation and the subsequent erosion of this impact crater. The clay-rich layers are the results of mid-Noachian processes, but they also formed after the martian dichotomy and possibly after intense erosion of earlier impact basins.

### 5.3. Standing bodies of water younger than the clay-bearing unit

If the origin of the clay-bearing unit remains uncertain from our orbital investigations, the observations of a fan-shaped feature, which we interpret to be a delta, provide more evidence for standing bodies of water. Observations of channels stratigraphically below the fan demonstrate different base levels (Fig. 12). If we accept that the current topography is broadly representative of the ancient landscape, the body of water was probably open to the northern lowlands, and hence an ocean or sea was occupying what is now the entire Chryse Planitia region. About 1300 km to the west another delta at a similar elevation, opening onto the Chryse Planitia and associated with similar rounded buttes, is found at the termination of the Hypanis Valles fluvial system (Fawdon *et al.*, 2018). This reinforces the idea that a large standing body of water occupied Chryse Planitia. The existence of a late Noachian/Early Hesperian ocean (Turbet and Forget, 2019), younger than the clay-rich unit era, will be a key hypothesis to test on the surface with the instrument suites of the ExoMars rover.

In the stratigraphy of the deltaic deposits, we have found interbedded craters that provide evidence for intermittent activity. We also have evidence of changes in water level with time. The rocks at Oxia Planum may have recorded the intermittent and decreasing late Noachian/early Hesperian oceanic activity, but it is difficult to determine precise timings when using orbital data alone. It is possible that the clay-bearing unit itself belongs to the same history of a decreasing ocean water level, and a change in the geochemistry from the formation of clays to opaline silica. If the materials that compose the clay-bearing unit were altered by the presence of an ocean, this would imply that the landscape was filled with water, even up to the elevation of Mawrth Vallis to the east, which hosts similar morphologies and suites of clay minerals. This would mean that the standing body of water at Oxia Planum was deep. As the delta-fan units likely represent a shallow marine setting, the rocks at Oxia Planum may record both relatively deep and shallow marine settings. However, until we explore the region *in situ*, these interpretations must remain as working hypotheses. An alternative scenario would be the alteration of the clay-rich unit by the diagenesis of fine-grain basaltic sediments by reaction with groundwater after deposition and during burial as documented in Gale crater (Rampe *et al.*, 2020). From our orbital investigation, all scenarios remain possible.

### 5.4. Suitability of Oxia Planum for ExoMars objectives

Several units present in Oxia Planum demonstrate that the site meets the scientific requirements of an ExoMars Rover landing site. The site exposes two distinct Noachian units that are related to aqueous processes: (1) 50–100 m of layered and altered clay-bearing rocks, and (2) deposits linked to the fluviodeltaic system, postdating the widespread clay-rich layered unit, indicating a later period of surface water and probably even ponded water in large quantities. Furthermore, the intense erosion underwent by the Oxia Planum region, especially the emplacement and slow removal of the dark resistant unit, suggests that many of the Noachian sediments here will have been protected from the martian environment for much of the time since they were emplaced.

Deciphering the formation environments for such diverse Noachian rocks will fulfill the requirement of the ExoMars Rover. Moreover, the scientific targets are well distributed within the large landing site ellipse. Apart from the exposures of the dark resistant unit, wherever the rover will land, it will be landing on the exposures of Noachian deposits related to the aqueous activity. More than 70% of the landing site ellipse corresponds to the clay-bearing unit, as mapped by spectral criteria with both CRISM and OMEGA data (Carter *et al.*, 2016). Given that the probability of landing is higher at the center of the landing ellipses, the chance of landing on clay-rich material is even higher, as the ellipses are centered on a large exposure of clay-bearing material.

Clay minerals are known to provide catalytic surfaces that can facilitate the synthesis of some organic compounds needed for the emergence of life (*e.g.*, Hazen and Sverjensky, 2010). The alkaline and reducing geochemical conditions in which Fe/Mg-phyllosilicates mainly form are also favorable for the transition from prebiotic to biotic activity (*e.g.*, Pinnavaia, 1983). On early Earth, clay minerals were probably ubiquitous at the surface, and seem to have played a crucial role in the origin and early evolution of life (Hazen and Sverjensky, 2010). Moreover, clay minerals on Earth are known to provide an impermeable barrier favorable for the preservation of biosignatures (Farmer and Des Marais, 1999). The pervasive clay-bearing rocks cropping out across the Oxia Planum landing site could have preserved any ancient biosignatures on Mars that formed or collected here (Poulet *et al.*, 2009). The high concentration of clays here further enhances the potential for preservation (Farmer and Des Marais, 1999). From the analysis of hyperspectral data, there is no evidence of low-grade metamorphic minerals such as chlorite, prehnite, or micas, which would indicate thermal processes that could degrade putative biosignatures. These two distinct aqueous environments are recorded in sedimentary deposits. Sediments are known to concentrate organic matter, especially in the case of a large catchment area toward a sink.

A concern is that Oxia rocks are >4 Gy old and organic matter is known to degrade in <100 Ma (*e.g.*, Farley *et al.*, 2014). However, we can expect that the continuous erosion rates suggested by inverted landform morphologies and the size distribution of impact craters protected the rocks currently exposed from long-term cosmic ray exposure. With the current crater obliteration rate from crater statistics on Oxia as being ∼8 cm/Ma, we can expect to remove ∼8 m every 100 Ma. The shielding of rock from long cosmic ray exposure is a significant scientific advantage as the Rover payload is partly dedicated to the search for organic matter. Thus, the recent exposition age of the phyllosilicate-rich surfaces is a good argument for biosignature preservation from cosmic rays.

## 6. Conclusion

Oxia Planum exhibits a rich geological history and provides abundant targets to meet the ExoMars Rover science objectives. First, Oxia Planum exposes outcrops of Noachian fractured layered and clay-bearing deposits over hundreds of kilometers of terrain, meaning that the rover has a high probability of landing on these terrains. These clay detections indicate that liquid water was in contact with the rocks and provide a means of biosignature preservation. Later, a standing body of water probably covered the site, allowing the formation of a delta fan with hydrated silica-bearing deposits. Both these environments provide ways to meet the ExoMars rover goal of searching for ancient life.

As the site was later covered in resistant material—which we suggest was of a volcanic origin—during the early Amazonian, biosignatures would have been preserved by this burial process. Since then, the site has undergone erosion, which has resulted in the re-exposure of these Noachian rocks. Oxia Planum may be a perfect place by which to decipher the formation environments for such diverse and ancient Noachian altered rocks in a setting where they have been recently exhumed. Oxia will be the oldest location thus far explored on Mars and fulfills the goals of the ExoMars Rover mission.

## Supplementary Material

Supplemental data

Supplemental data

Supplemental data

Supplemental data

## Abbreviations Used

CRISM: Compact Reconnaissance Imaging Spectrometer for Mars
CTX: Context Camera
DTM: Digital Terrain Model
ESA: European Space Agency
GIS: Geographic Information System
HiRISE: High Resolution Imaging Science Experiment
HRSC: High Resolution Stereo Camera
LPC: launch period closing
LPO: launch period opening
MOLA: Mars Orbiter Laser Altimeter
MSP: multispectral survey
OMEGA: Observatoire pour la Mineralogie, l'Eau, les Glaces et l'Activité
PDS: Planetary Data System
TES: Thermal Emission Spectrometer
THEMIS: Thermal Emission Imaging System

## Notes

Associate Editor: Jack Mustard
