# Supplementary material for: Oxia Planum: The Landing Site for the ExoMars “Rosalind Franklin” Rover Mission: Geological Context and Prelanding Interpretation
Source: Astrobiology. 2021 Mar 10;21(3):345–66. doi: 10.1089/ast.2019.2191 (PMC7987365; doi:10.1089/ast.2019.2191)
Supplement: Supplemental data [file Supp_Table1.docx]

Supplementary material

| Image type | HRSC | CTX | HiRISE |
| --- | --- | --- | --- |
| Image ID | h3081_0000  h3037_0000  h3059_0000 | B01_009880_1977_XN_17N024W  B03_010869_1961_XN_16N024W  B06_011937_1971_XN_17N023W  B07_012214_1970_XN_17N023W  D01_027642_1972_XI_17N025W  D02_028130_1972_XI_17N025W  D04_028631_1959_XN_15N023W  D04_028776_1959_XN_15N023W  D05_029264_1967_XI_16N025W  F03_036780_1989_XN_18N025W  G01_018662_1981_XN_18N022W  G02_018873_1976_XN_17N023W  G02_019084_1991_XI_19N024W  G03_019374_1991_XI_19N024W  G04_019651_1979_XN_17N024W  G09_021629_1996_XN_19N026W  G18_025097_1973_XN_17N026W  G20_026020_1982_XN_18N025W  G21_026376_1962_XN_16N024W  G21_026442_1968_XN_16N026W  G22_026864_1986_XN_18N027W  G23_027009_1981_XN_18N025W  P04_002694_1984_XI_18N024W  P06_003195_1980_XI_18N024W  P07_003894_1976_XI_17N025W  P12_005740_1974_XI_17N023W  P15_007019_1978_XN_17N024W  P22_009735_1977_XN_17N024W | ESP_011937_1970_RED  ESP_012715_1975_RED  ESP_019084_1980_RED  ESP_025097_1975_RED  ESP_026442_1970_RED  ESP_028130_1975_RED  ESP_028631_1965_RED  ESP_029264_1970_RED  ESP_036780_1985_RED  ESP_036925_1985_RED  ESP_037070_1985_RED  ESP_037136_1985_RED  ESP_037347_1985_RED  ESP_037558_1985_RED  ESP_037703_1980_RED  ESP_039154_1985_RED  ESP_039299_1985_RED  ESP_039721_1980_RED  ESP_039932_1980_RED  ESP_040077_1980_RED  ESP_040288_1980_RED  ESP_040433_1985_RED  ESP_040921_1985_RED  ESP_041066_1985_RED  ESP_041132_1985_RED  ESP_041211_1980_RED  ESP_041422_1985_RED  ESP_041989_1980_RED  ESP_042134_1985_RED  ESP_042345_1985_RED  ESP_042556_1985_RED  PSP_002694_1985_RED  PSP_003195_1985_RED  PSP_003894_1975_RED  PSP_005740_1970_RED  PSP_007019_1980_RED  PSP_009735_1985_RED  PSP_009880_1985_RED |

Supplementary Table S1. list of images used in this paper. For HiRISE images, both RED and COLOR product were used (here, only the RED products are listed for clarity).
