# Supplementary material for: Oxia Planum: The Landing Site for the ExoMars “Rosalind Franklin” Rover Mission: Geological Context and Prelanding Interpretation
Source: Astrobiology. 2021 Mar 10;21(3):345–66. doi: 10.1089/ast.2019.2191 (PMC7987365; doi:10.1089/ast.2019.2191)
Supplement: Supplemental data [file Supp_Fig1.docx]

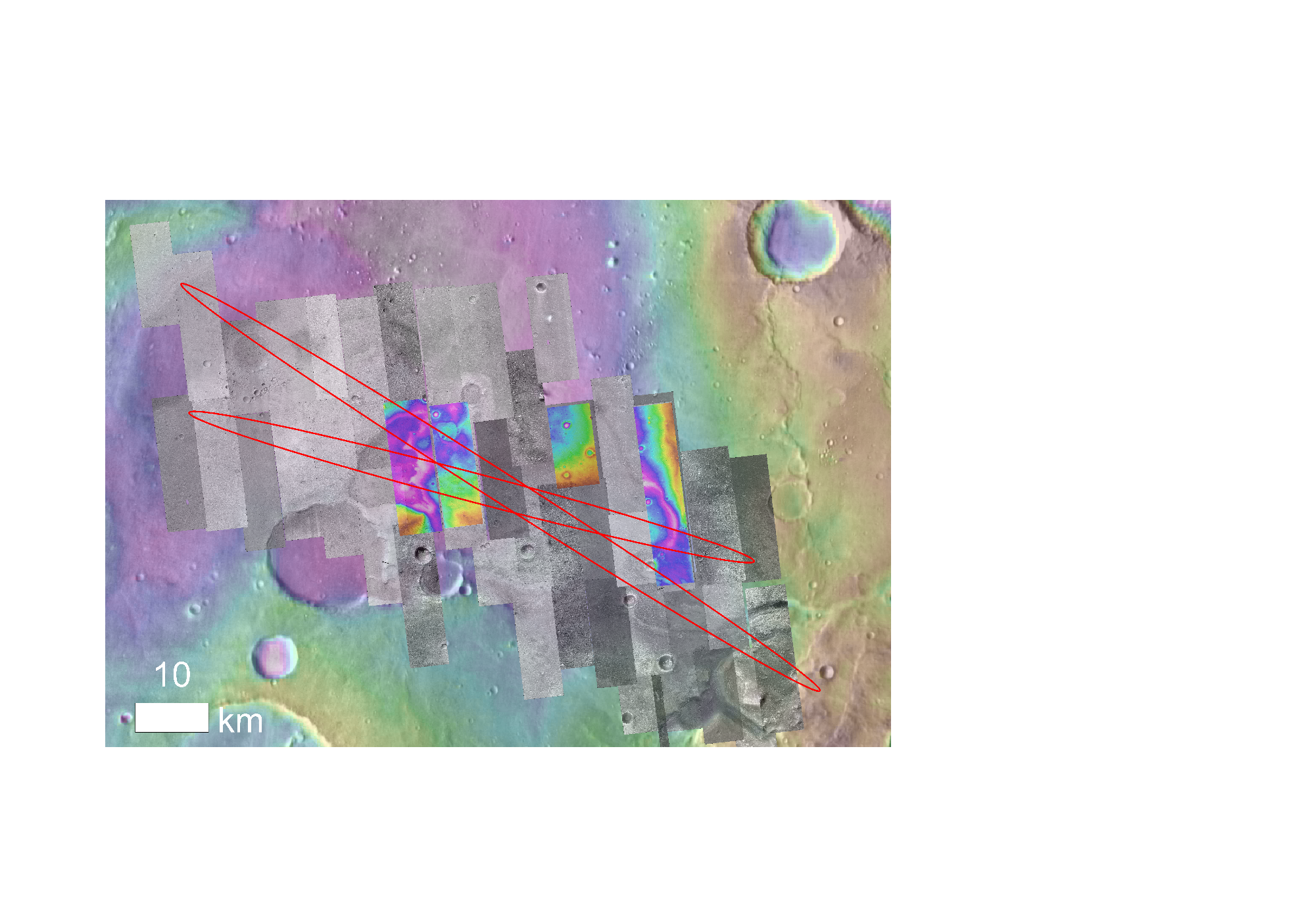


Supplementary Figure S1. HiRISE image coverage of ExoMars2020 ellipses and HiRISE DTM coverage used in this paper. Background image is MOLA topography superimposed on THEMIS day-time mosaics.
