# Supplementary material for: Oxia Planum: The Landing Site for the ExoMars “Rosalind Franklin” Rover Mission: Geological Context and Prelanding Interpretation
Source: Astrobiology. 2021 Mar 10;21(3):345–66. doi: 10.1089/ast.2019.2191 (PMC7987365; doi:10.1089/ast.2019.2191)
Supplement: Supplemental data [file Supp_Table2.docx]

| DTM type | HRSC | CTX | HiRISE |
| --- | --- | --- | --- |
| DTM ID or stereo pair ID | h3081_0000  h3037_0000  h3059_0000 | CTX_002694_1984_003195_1980  CTX_002694_1984_037070_1980  CTX_002694_1984_037136_1977  CTX_002694_1984_040433_1983  CTX_002694_1984_042134_1982  CTX_002694_1984_043413_1981  CTX_002694_1984_044679_1983  CTX_002694_1984_045800_1983  CTX_003195_1980_007019_1978  CTX_003195_1980_019651_1979  CTX_003195_1980_037070_1980  CTX_003195_1980_037136_1977  CTX_003195_1980_037703_1991  CTX_003195_1980_039299_1985  CTX_003195_1980_039721_1980  CTX_003195_1980_039932_1980  CTX_003195_1980_040077_1978  CTX_003195_1980_040433_1983  CTX_003195_1980_041422_1984  CTX_003195_1980_042134_1982  CTX_003195_1980_043268_1980  CTX_003195_1980_043413_1981  CTX_003195_1980_044679_1983  CTX_003195_1980_045167_1983  CTX_003195_1980_045800_1983  CTX_003894_1976_042622_1981  CTX_005740_1974_045523_1981  CTX_007019_1978_019084_1991  CTX_007019_1978_019374_1991  CTX_007019_1978_037070_1980  CTX_007019_1978_037136_1977  CTX_007019_1978_039721_1980  CTX_007019_1978_040077_1978  CTX_007019_1978_041211_1978  CTX_007019_1978_041989_1979  CTX_007019_1978_044824_1982  CTX_007019_1978_044890_1981  CTX_009735_1977_036925_2000  CTX_009735_1977_037558_2001  CTX_009735_1977_039154_1984  CTX_009735_1977_040921_1983  CTX_009735_1977_044811_1985  CTX_009735_1977_044956_1984  CTX_009880_1977_036925_2000  CTX_009880_1977_037558_2001  CTX_009880_1977_039154_1984  CTX_009880_1977_040921_1983  CTX_009880_1977_044811_1985  CTX_018873_1976_019374_1991  CTX_018873_1976_039721_1980  CTX_018873_1976_041211_1978  CTX_018873_1976_042345_1982  CTX_018873_1976_042846_1982  CTX_018873_1976_043558_1987  CTX_018873_1976_044824_1982  CTX_018873_1976_044890_1981  CTX_018873_1976_045101_1981  CTX_019084_1991_019374_1991  CTX_019084_1991_037136_1977  CTX_019084_1991_044824_1982  CTX_019084_1991_045101_1981  CTX_019374_1991_037070_1980  CTX_019374_1991_037136_1977  CTX_019374_1991_037703_1991  CTX_019374_1991_039721_1980  CTX_019374_1991_039932_1980  CTX_019374_1991_040077_1978  CTX_019374_1991_041211_1978  CTX_019374_1991_041422_1984  CTX_019374_1991_042345_1982  CTX_019374_1991_043268_1980  CTX_019374_1991_043413_1981  CTX_019374_1991_044046_1984  CTX_019374_1991_044257_1981  CTX_019374_1991_044890_1981  CTX_019374_1991_045101_1981  CTX_019651_1979_037136_1977  CTX_019651_1979_039299_1985  CTX_019651_1979_040077_1978  CTX_019651_1979_040433_1983  CTX_019651_1979_041989_1979  CTX_019651_1979_042134_1982  CTX_019651_1979_043268_1980  CTX_019651_1979_044679_1983  CTX_019651_1979_045800_1983  CTX_026020_1982_044468_1986  CTX_036780_1989_040921_1983  CTX_036780_1989_042622_1981  CTX_036780_1989_044178_1988  CTX_036925_2000_037558_2001  CTX_036925_2000_039299_1985  CTX_036925_2000_040433_1983  CTX_036925_2000_044679_1983  CTX_036925_2000_044811_1985  CTX_036925_2000_045800_1983  CTX_036925_2000_046156_1965  CTX_037070_1980_037136_1977  CTX_037070_1980_037703_1991  CTX_037070_1980_044046_1984  CTX_037070_1980_044257_1981  CTX_037070_1980_044824_1982  CTX_037136_1977_037703_1991  CTX_037136_1977_039721_1980  CTX_037136_1977_039932_1980  CTX_037136_1977_040077_1978  CTX_037136_1977_041422_1984  CTX_037136_1977_041989_1979  CTX_037136_1977_042134_1982  CTX_037136_1977_043268_1980  CTX_037136_1977_044046_1984  CTX_037136_1977_044257_1981  CTX_037136_1977_044824_1982  CTX_037136_1977_044890_1981  CTX_037347_1983_040921_1983  CTX_037347_1983_042622_1981  CTX_037347_1983_043690_1986  CTX_037347_1983_044178_1988  CTX_037558_2001_039154_1984  CTX_037558_2001_039299_1985  CTX_037558_2001_042701_1981  CTX_037558_2001_044679_1983  CTX_037558_2001_044811_1985  CTX_037558_2001_044956_1984  CTX_037558_2001_046156_1965  CTX_037703_1991_039721_1980  CTX_037703_1991_041211_1978  CTX_037703_1991_042345_1982  CTX_037703_1991_043413_1981  CTX_037703_1991_044824_1982  CTX_037703_1991_044890_1981  CTX_037703_1991_045101_1981  CTX_039154_1984_040921_1983  CTX_039154_1984_044811_1985  CTX_039154_1984_046156_1965  CTX_039299_1985_040433_1983  CTX_039299_1985_042701_1981  CTX_039299_1985_045167_1983  CTX_039299_1985_045800_1983  CTX_039721_1980_043558_1987  CTX_039721_1980_044824_1982  CTX_039721_1980_045101_1981  CTX_039721_1980_045378_1981  CTX_039932_1980_040433_1983  CTX_039932_1980_043413_1981  CTX_039932_1980_044824_1982  CTX_040077_1978_044824_1982  CTX_040433_1983_042701_1981 | DTM N°1 from ESP_037558_1985 and  ESP_036925_1985  DTM N°2 from PSP_003195_1985 and PSP_002694_1985  DTM N°3 from ESP_009735_1985  and ESP_009880_1985  DTM n°4 from  ESP_037136_1985 and  ESP_ 037070_1985 |

Supplementary Table S2. list of DTMs used in this paper. HRSC IDs are from the ESA server; CTX DTM IDs are generated by the MarsSI facility (Quantin-Nataf et al., 2018) and joined both ID of the two used CTX stereopairs; HiRISE DTM have been numbered from 1 to 4 after their creation date.
