# Supplementary material for: Oxia Planum: The Landing Site for the ExoMars “Rosalind Franklin” Rover Mission: Geological Context and Prelanding Interpretation
Source: Astrobiology. 2021 Mar 10;21(3):345–66. doi: 10.1089/ast.2019.2191 (PMC7987365; doi:10.1089/ast.2019.2191)
Supplement: Supplemental data [file Supp_Fig2.docx]

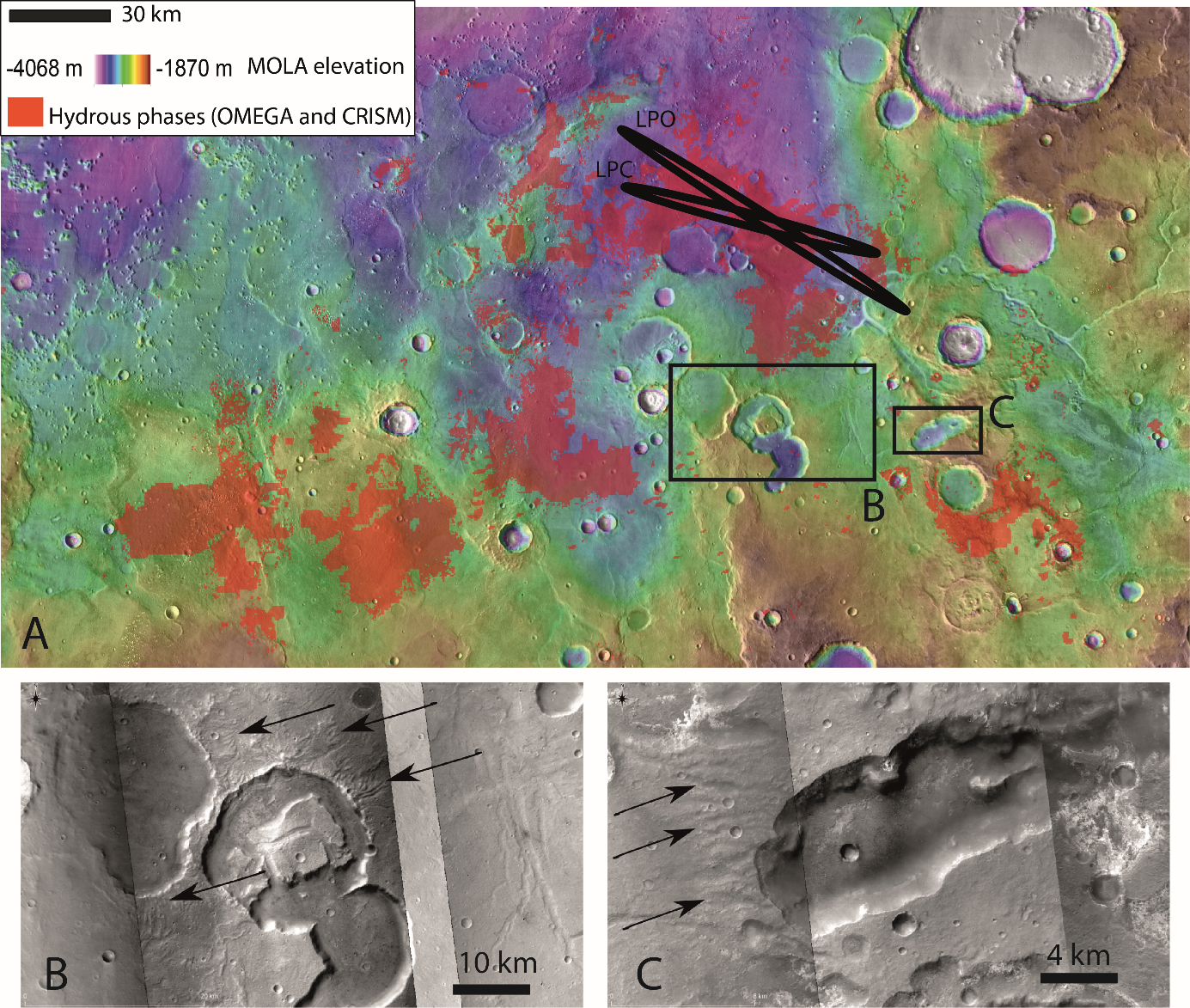


Supplementary Figure S2. Putative volcanic domes : A] Topographic and mineralogical context presented in Figure 3 and location of the subfigures B and C. B] Putative volcanic dome with flanks etched by several quasi-radial furrows starting directly at the rim of the submittal depression. D] Putative non-circular volcanic domes with flanks etched by several quasi-radial furrows starting directly at the rim of the submittal depression.
